# MRI abdominal fat segmentation with a novel VAT/SAT delineation algorithm: Otsu regression calibration and comparison to k-means and fuzzy c-means

**DOI:** 10.3389/fbinf.2026.1752522

**Published:** 2026-05-14

**Authors:** Mihai Octavian Negrea, Bogdan Neamtu, Denisa Claudia Roman, Darius Peteleaza, Ciprian Sofariu, Raluca Maria Costea, Olga Brusnic

**Affiliations:** 1 Faculty of Medicine, Lucian Blaga University of Sibiu, Sibiu, Romania; 2 Pediatric Research Department, Pediatric Clinical Hospital of Sibiu, Sibiu, Romania; 3 Whiting School of Engineering, Johns Hopkins University, Baltimore, MD, United States; 4 Computer Science and Electrical Engineering Department, Faculty of Engineering, Lucian Blaga University of Sibiu, Sibiu, Romania; 5 Internal Medicine Department, George Emil Palade University of Medicine, Pharmacy, Science, and Technology of Târgu Mureș, Targu Mures, Romania; 6 Gastroenterology Department, Mures County Clinical Hospital, Targu Mures, Romania

**Keywords:** abdominal fat segmentation, fuzzy c-means clustering, k-means clustering, Otsu thresholding, somatic adipose tissue, statistical analysis, visceral adipose tissue

## Abstract

**Introduction:**

Obesity is a major cardiovascular risk factor, particularly in excessive visceral adipose tissue accumulation. This study assessed the efficiency of three image processing algorithms (Otsu, K-means, and Fuzzy C-means) in quantifying abdominal adipose tissue from single-slice MRI image analysis obtained using a standard acquisition protocol.

**Methods:**

We developed a novel, open-source algorithm to delineate visceral and somatic adipose tissue by comparing the distance from the centroid of the largest segmented regions to the center of the image. Segmentation methods were evaluated against a manual reference.

**Results:**

The study included 68 patients (30 males and 38 females) aged between 8 and 84. All algorithms showed satisfactory accuracy, with Otsu thresholding consistently performing slightly better. Segmentation efficiency was analyzed within subgroups defined by gender, age, weight status, and diagnosis. Accuracy remained acceptable across subgroups, although male sex and higher weight status in adults were associated with superior results. Linear regression models were implemented to address errors concerning visceral and somatic adipose tissue quantification. Correlations between adipose tissue surface and BMI emerged, with significant differences in adipose tissue distribution across genders and age groups.

**Discussion:**

Our findings highlight the potential of MRI-based adipose tissue segmentation in cardiovascular risk stratification using a novel, open-source algorithm.

## 1 Introduction

Obesity has been extensively recognized as a cardiovascular risk factor (Koliaki et al., 2019). The excessive accumulation of visceral adipose tissue (VAT) is known to have adverse impacts on cardiometabolic health, as outlined in a comprehensive literature review we have conducted (Negrea et al., 2021). Various techniques have been explored to quantify visceral adipose tissue, with computed tomography being acknowledged as the gold standard (Wajchenberg, 2000; Fourman et al., 2019; Zemel, 2011; Huang et al., 2001). Its use is limited, however, due to radiation exposure, especially in pediatric patients. Magnetic resonance imaging (MRI) has emerged as a viable alternative demonstrating comparable results (Klopfenstein et al., 2012; Simoni et al., 2020; Pescatori et al., 2019; Zaffina et al., 2022). Several methods of quantifying abdominal visceral adipose tissue have been described using MRI (Pescatori et al., 2019; Shen et al., 2004a; Shen et al., 2004b; Shen et al., 2005; Shen et al., 2012; Uthaya et al., 2004; Samara et al., 2012; Brown et al., 2015; Eloi et al., 2017; Murata et al., 2022; Hu et al., 2011; Positano et al., 2004; Marunowski et al., 2021; Poonawa et al., 2013; Linder et al., 2020; Schaudinn et al., 2015; Schwenzer et al., 2010; Springer et al., 2012; Storz et al., 2018; Bonekamp et al., 2008; Ross et al., 1996; Thörmer et al., 2013). Image manipulation generally involves pre-processing followed by a method of discriminating between adipose and lean tissue against a background (Eloi et al., 2017; Positano et al., 2004). Examples in this respect include thresholding, fuzzy C-means clustering, or k-means clustering (Pescatori et al., 2019; Uthaya et al., 2004; Positano et al., 2004; Ross et al., 1996; Gerber and Peterson, 2008; Bernecker et al., 2018; Dougherty and Lotufo, 2003; Lim, 1990; Gonzalez and Woods, 2018; Ogawa et al., 2020; Otsu, 1979; Chaudry et al., 2020; Xing et al., 2020; Thörmer et al., 2013; Würslin et al., 2010; Choudhry and Kapoor, 2016; Makrogiannis et al., 2012), which are briefly described in the supplementary information. The distinction between subcutaneous and visceral adipose tissue can be achieved by either manual (Pescatori et al., 2019; Poonawa et al., 2013), semiautomated (Shen et al., 2005; Poonawa et al., 2013), or automated processes (Positano et al., 2004; Thörmer et al., 2013; Würslin et al., 2010). While MRI-based adipose tissue quantification software packages are available (Pescatori et al., 2019; Shen et al., 2004a; Shen et al., 2004b; Shen et al., 2012; Uthaya et al., 2004; Eloi et al., 2017; Murata et al., 2022; Positano et al., 2004; Marunowski et al., 2021; Poonawa et al., 2013; Linder et al., 2020; Schaudinn et al., 2015; Schwenzer et al., 2010; Springer et al., 2012; Storz et al., 2018; Bonekamp et al., 2008; Thörmer et al., 2013) (see Supplementary Table S1), many require manual intervention, paid subscriptions, or third-party requests. Even though free alternatives exist, they lack validation in large studies (Maddalo et al., 2017). AI-assisted models show promise but often require large training datasets (Langner et al., 2019). In addition, a consensus is yet to be reached regarding the optimal acquisition protocol and localization for abdominal tissue quantification. Single-slice approaches provide reproducible, time-saving, and cost-effective methods. Significant efforts have been made to distinguish the ideal position for analysis (Pescatori et al., 2019; Shen et al., 2004a; Shen et al., 2004b; Brown et al., 2015; Eloi et al., 2017; Linder et al., 2020; Schaudinn et al., 2015; Schwenzer et al., 2010; Shen et al., 2007) (examples provided in Supplementary Table S2). Growing data suggest, however, that the L2 position might provide optimal results when predicting cardiometabolic effects associated with visceral adipose tissue. This position has been validated in diverse populations (Positano et al., 2004; Marunowski et al., 2021; Schaudinn et al., 2015; Shen et al., 2007; O’Connor et al., 2015; Lee et al., 2011; Kuk et al., 2010; K et al., 2006).

Furthermore, there is a lack of consensus regarding MRI acquisition protocols (Pescatori et al., 2019; Shen et al., 2004a; Shen et al., 2004b; Shen et al., 2012; Uthaya et al., 2004; Brown et al., 2015; Eloi et al., 2017; Murata et al., 2022; Positano et al., 2004; Marunowski et al., 2021; Linder et al., 2020; Schwenzer et al., 2010; Springer et al., 2012; Thörmer et al., 2013; Würslin et al., 2010) (see Supplementary Table S3 for frequently employed parameters). Among the more frequently implemented protocols, gradient echo in-phase/opposed-phase sequences are T1-weighted image acquisition techniques that exploit the differences in resonance frequencies between the protons associated with fat and those associated with water (Earls and Krinsky, 1997). The different cyclic variation of resonance frequencies of these two types of protons is determined by the influence of adjacent atom structures, resulting in water and fat signals periodically registering in phase with each other, thus enhancing the signal additively, or in opposite phases, effectively canceling each other out (Earls and Krinsky, 1997; Outwater et al., 1998). This leads to a loss in signal at the interface between tissues containing fat and water, known as the India-ink artifact (Bennett and Dyer, 2017). Common implementations of the technique include the diagnosis of various abdominal tumors or the presence of hepatic steatosis (Outwater et al., 1998), an entity linked to metabolic syndrome and visceral adipose tissue surplus (Chalasani et al., 2012; Lemieux and Després, 2020; Di et al., 2014). In addition, the clear visualization of interfaces between fat- and water-rich tissues in opposed-phase MRI provides a basis for a precise delineation between fat and lean mass, thus enabling efficient abdominal tissue segmentation (Thörmer et al., 2013).

Our study aimed to compare the efficiency of image thresholding using the Otsu method, K-means, and fuzzy C-means algorithms in abdominal adipose tissue quantification, assessing performance against a manual reference obtained via a technique that has been previously validated (Eloi et al., 2017; Maddalo et al., 2017). These algorithms were combined with a novel method concerning automatic differentiation between visceral and somatic adipose tissue. The goal was to identify the most efficient algorithm that could provide satisfactory results regardless of patients’ demographics, weight status, or diagnosis. We developed a freely accessible, open-source algorithm capable of automated adipose tissue segmentations using single-slice analysis from a commonly implemented MRI protocol in clinical practice. In addition, the demographic and anthropometric variables that could influence the accuracy of the implemented methods were explored.

## 2 Materials and methods

### 2.1 Study setting and population

We conducted a retrospective study of abdominal MRI examinations retrieved from the clinical database of the Pediatric Clinical Hospital of Sibiu, Romania, between 11 November 2015 and 16 May 2023. The database was last accessed for data acquisition on 17 May 2023. The study adhered to the Declaration of Helsinki and was approved by the Institutional Review Committee of the Pediatric Clinical Hospital of Sibiu (registration number 6758/05.10.2021). Participants provided informed consent for use of their data for purposes including medical education and research according to institutional personal data processing protocols; for minors, consent was obtained from parents or legal guardians.

The study cohort included patients aged 8 years and older, irrespective of weight status or diagnosis. We excluded patients with extensive prior abdominal surgery or significant space-occupying intra-abdominal pathology; repeated examinations of the same patient; studies lacking in-/opposed-phase images; and slices with heavy motion artifacts or suboptimal field of view (FOV) for adipose tissue quantification. Examinations without a slice at the L2 vertebral level (either entirely or due to apparent subcutaneous adipose tissue (SAT)-VAT communications) were excluded, as were examinations in which abundant bowel content could be misclassified as adipose tissue. Patients with missing data and those who did not consent to scientific use of their data were not included.

Demographic (age, sex) and anthropometric data (weight, height), and the primary diagnosis after MRI interpretation were extracted. Per institutional protocol, height is recorded to the nearest 0.5 cm and weight to the nearest 0.1 kg on a calibrated electronic scale before each examination. Body mass index (BMI) was calculated according to World Health Organization guidance (World Health Organization, 2025a). BMI z -scores for children (5–19 years) were computed using the WHO 2007 growth reference (LMS method) and the accompanying documentation/tables (World Health Organization, 2025b). WHO criteria for childhood overweight and obesity were applied: overweight >1 SD and obesity >2 SD above the BMI-for-age median (World Health Organization, 2025c). For adults, overweight/obesity were defined using CDC BMI cut-offs (Centers for D isease Control and Prevention, 2024).

### 2.2 MRI acquisition

#### 2.2.1 Acquisition protocol characteristics

We included studies with T1 gradient-echo in-/opposed-phase images (manually set FOV; acquisition matrix 256×176 ; TR 124; TE 2.46; slice thickness 5 mm; interslice spacing 6.5 mm). For each patient, a single axial slice was selected at the L2 level with clear tissue interfaces. Images were exported as TIFF files (512×352) for processing.

#### 2.2.2 L2 plane identification

The L2 vertebral level was identified on sagittal localizer images using a technique previously described in the literature based on initial L5 identification (Hughes and Saifuddin, 2006). The L5 vertebra was localized by identifying the iliolumbar ligaments on axial and/or sagittal images, which are recognized anatomical landmarks typically originating from the transverse process of L5 and inserting into the iliac crest. This method has been described as a practical and reproducible approach for lumbar vertebral numbering in MRI studies (Jagannathan et al., 2017). After confirming L5, vertebral bodies were counted cranially to determine the L2 level. For quantitative analysis, the axial slice selected for segmentation corresponded to the image located closest to the craniocaudal midpoint of the L2 vertebral body, as determined on sagittal reconstructions.

### 2.3 Image processing

#### 2.3.1 Study workflow

**FIGURE 1:**
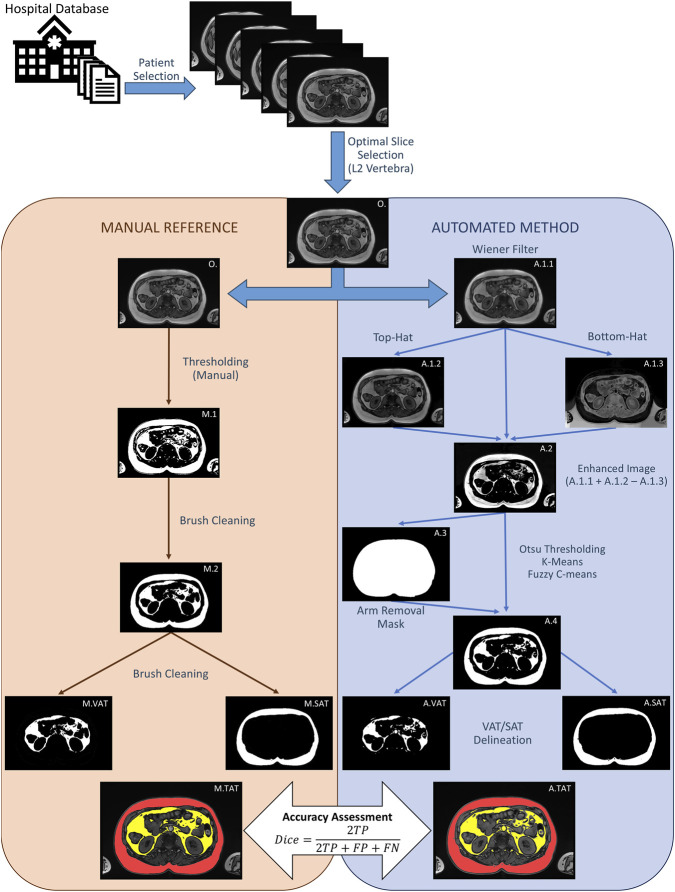
Study workflow.

Figure 1 shows the overall study workflow in a representative patient, beginning with case selection from the hospital database according to the inclusion and exclusion criteria described in Section 2.1, followed by identification of the optimal axial slice at the L2 vertebral level on sagittal localizer images, as detailed in Section 2.2. The selected slice was then processed through two parallel pipelines: manual reference segmentation (left panel, orange) and automated segmentation (right panel, blue). In the manual workflow, the original image (O) underwent intensity thresholding to generate an initial binary adipose tissue mask (M.1), as described in Section 2.4, which was then manually refined using the brush tool to remove misclassified non-adipose structures and produce the total adipose tissue mask (M.2). Separate subcutaneous adipose tissue (M.SAT) and visceral adipose tissue (M.VAT) masks were subsequently generated by manual removal of the complementary compartment, and the final composite image (M.TAT) shows the original image with manually delineated SAT and VAT overlaid in red and yellow, respectively.

In the automated workflow, images first underwent preprocessing as described in Section 2.3, including Wiener filtering (6 × 6) for denoising (A.1.1) and top-hat (A.1.2) and bottom-hat (A.1.3) morphological transformations to enhance adipose–lean tissue contrast (Bouazizi et al., 2021); these were combined with the original image to generate the final preprocessed image (A.2), which served as input for segmentation. The preprocessed image was then segmented using Otsu thresholding, K-means clustering, and fuzzy C-means clustering, as described in Sections 2.3.1.1, 2.3.1.2, and Section 2.3.1.3. Before adipose tissue delineation, arm removal was performed by applying an arm-removal mask (A.3), and image A.4 shows an example of the Otsu-based segmentation result after mask application. VAT and SAT were then separated using the automated VAT/SAT delineation rule, and the final composite image (A.TAT) shows the automated segmentation overlaid on the original image, with SAT in red and VAT in yellow. Finally, quantitative surface measurements were calculated as described in Section 2.5, and the accuracy of each automated segmentation result was evaluated against the manual reference according to Section 2.6.

##### 2.3.1.1 Threshold (Otsu) method

The optimal threshold was computed via Otsu’s method and applied to the pre-processed slice to obtain a binary mask (scikit-image) (Otsu, 1979). 8-connected components (N, S, E, W, NE, NW, SE, SW) were labeled; region properties (area, pixel count, centroid) were measured and stored (scikit-image). The image center and each region’s centroid–center Euclidean distance were computed. Arm regions were removed (see below), and VAT/SAT delineation was performed from distances/areas as detailed below. Final SAT and VAT masks were saved to disk.

##### 2.3.1.2 K-means method

Images were flattened and reshaped for K-means (NumPy). With K=2 , K-means clustering (scikit-image) was applied; results were reshaped to image dimensions with background labeled “0” (black). Arm removal and VAT/SAT delineation followed the same procedure as for Otsu. Outputs were saved.

##### 2.3.1.3 Fuzzy C-means method

Fuzzy C-means followed the K-means pipeline with the addition of a fuzziness coefficient (scikit-fuzzy). Arm removal and VAT/SAT delineation were identical to the above.

##### 2.3.1.4 VAT/SAT delineation (novel rule)

We delineated VAT from SAT by comparing centroid–center distances among the largest connected adipose regions. The region further from the image center was labeled SAT (peripheral), and the region closer to center was labeled VAT (central). This rule, implemented in NumPy, was applied identically to all three segmentation outputs and provides a simple, generalizable criterion for VAT/SAT separation.

##### 2.3.1.5 Arm removal

An abdominal mask was generated to exclude upper-extremity subcutaneous fat. Pixels in the abdominal region were set to 1 (white) and multiplied with the image (logical AND) to suppress arms (scikit-image). The same mask was used for all automated methods.

### 2.4 Manual reference

Manual segmentation provided a reference standard. Using ImageJ, a validated manual thresholding workflow was applied to separate adipose from lean tissue and generate binary masks (Eloi et al., 2017). A total adipose tissue (TAT) mask was refined with the brush tool on opposed-phase images to remove lean tissue misclassified as fat, as in prior work (Maddalo et al., 2017). VAT and SAT masks were created by selectively erasing the complementary somatic/visceral compartments. Inter-observer agreement was assessed for quantitative measurements derived from manual segmentation performed by two experienced observers: a medical imaging specialist with more than 10 years of experience in pediatric and adult MRI interpretation and an experienced gastroenterologist (Linder et al., 2019; Abildgaard et al., 2021).

### 2.5 Surface calculations

TAT, SAT, and VAT areas (“surfaces”) were computed as the number of adipose pixels multiplied by pixel area, following prior practice (Cervantes et al., 2019). Pixel area was calculated as FOV divided by matrix sampling in the phase/frequency directions (Strzelecki et al., 2022; Mezrich, 1995). Age-adjusted reference intervals for children would ideally be used (Marunowski et al., 2021), but were unavailable for our protocol; therefore, adipose distribution was summarized via VAT/TAT (%) and VAT/SAT ratios, consistent with Storz et al. (Storz et al., 2018).

### 2.6 Evaluation of accuracy

Manual masks were loaded as ground truth alongside automated predictions. Inter-observer agreement for manual SAT and VAT surface measurements was high, with ICC(2,1) values of 0.8445 and 0.8415, respectively, supporting the reproducibility of the manual reference standard. We computed the confusion matrix (TP: adipose pixels correctly identified; TN: background/lean correctly identified; FP: background/lean mislabeled as adipose; FN: adipose mislabeled as background/lean). From these, the Dice coefficient and Hausdorff distance were computed. Given class imbalance, the Dice coefficient was the primary accuracy metric (Müller et al., 2022).

### 2.7 Statistical analysis

Analyses and visualizations were performed in Microsoft Excel and IBM SPSS Statistics. Correlations between continuous variables were examined using the Student’s t -test for independent, normally distributed variables, and the Mann–Whitney U test otherwise. Paired data were analyzed with the paired-samples t -test (normal) or the Wilcoxon signed-rank test (non-normal). For multiple groups, we used one-way ANOVA when normality held within groups or the Kruskal–Wallis test otherwise; repeated-measures ANOVA was used for within-subject designs. Normality was assessed using Shapiro–Wilk or Kolmogorov–Smirnov tests, skewness/kurtosis, and visual inspection of box plots and Q–Q plots. Categorical associations were assessed with Chi-square or Fisher’s exact tests.

Linear regression first assessed independent associations between predictors and the Dice coefficient (Otsu method) identified in univariate analyses. To account for heterogeneity, we performed bootstrapping with 10,000 resamples and bias-corrected accelerated confidence intervals. We then applied exhaustive forward–and backward selection to derive linear models for surface-quantification error corrections. Categorical predictors (sex, age category, obesity status) were dummy-coded. To correct systematic underestimation of Otsu-derived VAT and SAT areas and adjust for unequal Sex × Age subgroup sizes, we fitted weighted least-squares (WLS) regressions using inverse-frequency weights (reciprocal of subgroup counts) in Python. The VAT model regressed manual VAT on Otsu VAT, obesity status, age category, sex, and their interactions; the SAT model was specified analogously. Model fit was evaluated by $R^{2}$ and 95% CIs from WLS standard errors, and agreement by Bland–Altman limits.

Potential multicollinearity among predictors considered for the correction models was assessed using pairwise correlation matrices and variance inflation factors (VIFs) computed from the corresponding predictor design matrices. For models including interaction terms, binary predictors were mean-centered prior to interaction construction for VIF estimation.

**FIGURE 2:**
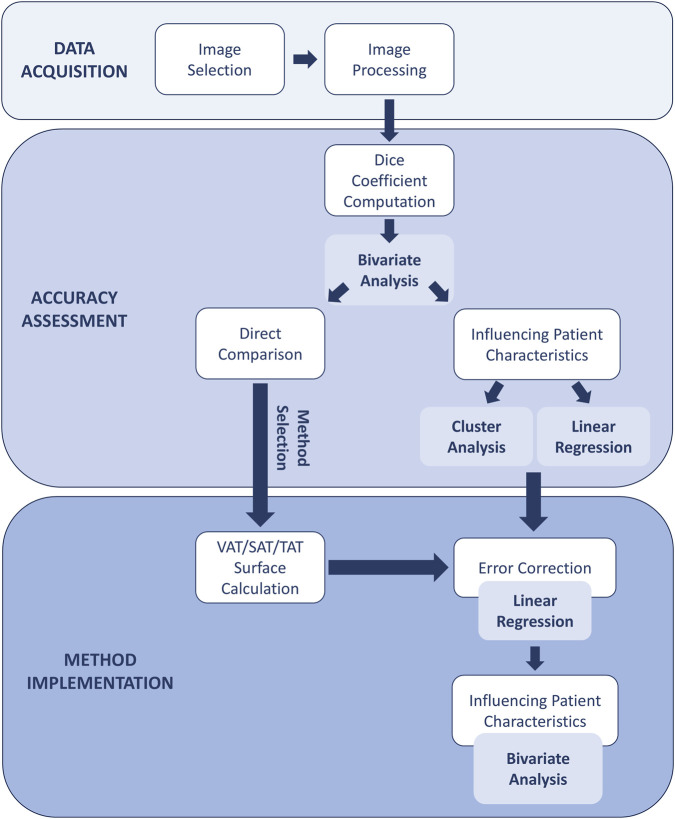
Data processing workflow.

Internal validation of the final correction models was performed by leave-one-out cross-validation and repeated 10-fold cross-validation (100 repeats), with model refitting and inverse-frequency Sex × Age weight recalculation performed within each training subset and evaluation on held-out cases. The analysis workflow is summarized in Figure 2.

## 3 Results

### 3.1 Study population demographics and characteristics

A total of 173 patients underwent abdominal MRI in our center between November 2015 and May 2023. Of these, 68 patients were included (30 males, 38 females). The exclusion criteria are listed in Supplementary Table S4.

Patient age ranged from 8 to 84 years (mean 40.69±22.18 years). Males were significantly younger than females ( 33.50±23.21 vs. 46.37±19.83 years; p=0.015 ). Obesity was more prevalent in children and in males. Regarding MRI interpretation, 17 patients had no discernible pathology, 8 had malignant conditions, and 43 had benign processes. Malignancies were more prevalent among adults and showed no correlation with weight status or gender. Diagnosis frequencies appear in Supplementary Figure S1. The demographic characteristics, distribution across weight status, and diagnosis type categories are presented in Table 1.

**TABLE 1:** Characteristics of included patients (% within row category).

Age group		Children	Adults	p-value
Gender	Male	15 (71.4%)	15 (31.9%)	<0.01
	Female	6 (28.6%)	32 (68.1%)	

Weight status		Normal weight	Overweight	Obesity	p-value
Gender	Male	5 (16.7%)	11 (36.7%)	14 (46.7%)	0.045
	Female	17 (44.7%)	8 (21.1%)	13 (34.2%)	
Age category	Children	5 (23.8%)	2 (9.5%)	14 (66.7%)	<0.01
	Adults	17 (36.2%)	17 (36.2%)	13 (27.7%)	

Diagnosis		Normal MRI	Benign	Malignant	p-value
Gender	Male	11 (36.7%)	16 (53.3%)	3 (10.0%)	0.173
	Female	6 (15.8%)	27 (71.1%)	5 (13.2%)	
Age category	Children	8 (38.1%)	13 (61.9%)	0 (0%)	0.048
	Adults	9 (19.1%)	30 (63.8%)	8 (17.0%)	
Weight status	Normal weight	4 (18.2%)	16 (72.7%)	2 (9.1%)	0.834
	Overweight	5 (26.3%)	12 (63.2%)	2 (10.5%)	
	Obese	8 (25.0%)	15 (55.6%)	4 (14.8%)	

### 3.2 Overall performance and method comparison

The overall performance of the algorithms, measured by the Dice coefficient, is shown in Table 2. Otsu thresholding consistently outperformed the K-means and Fuzzy C-means algorithms. Similarly, the K-means algorithm outperformed Fuzzy C-means in all instances. Despite being statistically significant, however, the differences in performance between algorithms were minimal.

**TABLE 2:** Performance (Dice coefficient; higher is better) of all three algorithms.

Tissue	Otsu	K-means	Fuzzy C-means	p -value
TAT	0.9152±0.0333	0.9099±0.0368	0.9082±0.0368	<0.01
SAT	0.9647±0.0150	0.9646±0.0150	0.9637±0.0152	<0.01
VAT	0.7863±0.1070	0.7856±0.1070	0.7807±0.1070	<0.01

To complement the Dice analysis, Table 3 reports boundary accuracy using the Hausdorff distance. Under this metric, Otsu achieves the lowest distances for TAT and VAT, while K-means is marginally best for SAT. Fuzzy C-means yields higher distances across all tissues. The Otsu–K-means differences are small for TAT and SAT but more pronounced for VAT. The overall pattern aligns with the Dice-based comparison: Otsu and K-means perform similarly, both outperforming Fuzzy C-means, with the largest separation observed for VAT.

**TABLE 3:** Performance (Hausdorff distance; lower is better) of all three algorithms**.**

Tissue	Otsu	K-means	Fuzzy C-means	p -value
TAT	10.7204±2.8020	10.7474±2.7441	10.7542±2.7389	<0.01
SAT	9.1969±3.0842	8.9252±2.9729	9.2111±3.0727	<0.01
VAT	7.8279±1.2473	9.6224±2.4703	10.0460±2.8553	<0.01

**FIGURE 3:**
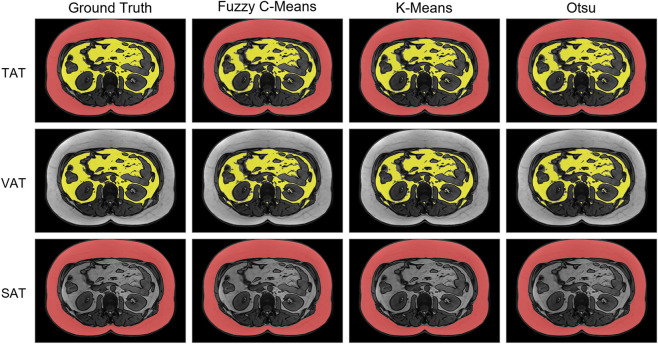
Example of a case with a strong model performance.

**FIGURE 4:**
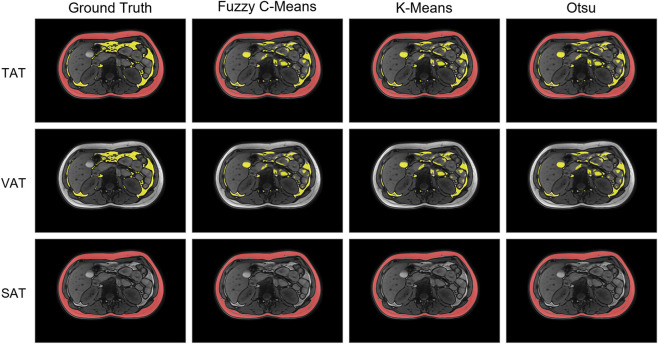
Example of a case with a weak model performance.

To improve the interpretability and visual impact of our study, we included two examples of model segmentation outputs illustrating strong and weak model performance (Figures 3, 4). The example of strong model performance yielded Dice coefficients of 0.9652 for TAT, 0.9858 for SAT, and 0.8186 for VAT, with corresponding Hausdorff distances of 7.8102, 6.6332, and 5.4772, respectively. In contrast, the example of weak model performance yielded Dice coefficients of 0.8475 for TAT, 0.9484 for SAT, and 0.6418 for VAT, with corresponding Hausdorff distances of 12.0416, 11.3137, and 10.6954, respectively.

### 3.3 Algorithm performance within patient groups

We compared the algorithms’ performance within specific patient groups across gender, age categories, weight status, and diagnosis type. The main results which apply to all algorithms are summarized in Table 4 (details in Supplementary Table S5).

**TABLE 4:** Performance of algorithms across patient groups.

Variable	Values	Higher dice coefficient for
Gender	Male > female	VAT, TAT
Age category	Children > adults	SAT
	Adults > children	VAT
Weight status	Obese > overweight > normal weight	TAT, SAT, VAT
Diagnosis type	—	No correlations found

### 3.4 Cluster analysis

We conducted a two-step cluster analysis to capture the intricate interactions among parameters that impact the precision of visceral adipose tissue quantification. We used Akaike’s information criterion based on the Dice values for visceral adipose tissue quantification obtained by using the Otsu method (as this method consistently delivered more accurate results in our study population), gender, age category, and dichotomized weight category (overweight/obese versus normal weight), allowing for automatic determination of cluster number. The model produced five clusters with an average silhouette of 0.6, indicating a good fit. Cluster attributes are detailed in Supplementary Table S6.

**FIGURE 5:**
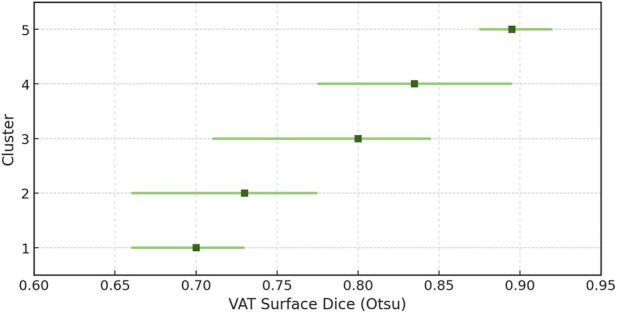
Distribution of Dice values across clusters.

The dice values progressively increased from cluster 1 to cluster 5; the distribution (median with 25th/75th percentiles) is shown in Figure 5. Cluster 5 primarily comprised adult overweight/obese males; Cluster 4, adult overweight/obese females; Cluster 3, overweight/obese male children; Cluster 2, normal-weight adult females; and Cluster 1, normal-weight female children.

### 3.5 Linear regression for accuracy predictors

We examined whether the variables that influenced the precision of the Otsu method exerted their influence independently while adjusting for the variances in gender distribution across weight and age categories. To this end, we applied linear regression analysis, with the Dice coefficient for VAT measurement based on the Otsu method selected as the outcome variable. Binary dummy variables were coded for gender, age category, and weight category. Females, childhood, and normal weight served as reference points. These variables were subsequently integrated as independent variables in the regression.

The overall regression model was statistically significant: $R^{2}=0.289$ ; F=8.683 ( ${\text{df}}_{\text{Regression}}=3$ ; ${\text{df}}_{\text{Residual}}=64$ ); p<0.01 . Thus, the selected variables were revealed to be autonomous predictors of the Otsu method’s efficacy (details can be found in Supplementary Table S7).

### 3.6 Agreement between methods and corrections

The automated methods deployed in this study demonstrated a statistically significant underestimation of both SAT and VAT values. Using the Otsu threshold method, VAT was consistently underestimated by a mean of $20.03\;{cm}^{2}$ (SD $14.06\;{cm}^{2}$ ) and SAT by $9.22\;{cm}^{2}$ (SD $5.81\;{cm}^{2}$ ). The differences were normally distributed in both cases. To compensate for these errors, we conducted linear regression analyses with the manually determined areas as dependent variables.

In the first model, the VAT surface area calculated by the manual reference method was chosen as the dependent variable. The independent variables were the VAT surface computed by the Otsu algorithm and the presence of obesity. The regression was statistically significant $\left(R^{2}=0.978;\;F=1465.649\;\left({\text{df}}_{\text{Regression}}=2;\;{\text{df}}_{\text{Residual}}=65\right);\;p<0.01\right)$ , and the fitted regression model was Equation 1.

$$\begin{array}{ll} {VAT}_{\text{manual}} & =7.224+1.112\times {VAT}_{\text{Otsu}}+7.583\times I_{\text{Obesity}}, \end{array}$$ (1)

where all areas are in ${cm}^{2}$ and $I_{\text{Obesity}}=1$ for obese patients and 0 otherwise. This method ensured appropriate adjustments were made to account for the observed underestimations. A more comprehensive description of the regression model is available in Supplementary Table S8. The model showed a good correlation with the manually determined VAT surface $\left(R^{2}=0.978\right)$ , as shown in Supplementary Figure S2. Bland–Altman analysis indicated that estimating the VAT surface by applying Equation 1 to the Otsu results provides limits of agreement (LoA) between −21.46 and $+21.51\;{cm}^{2}$ (Supplementary Figure S3). Refitting the WLS VAT model on the full cohort (n=68) yielded: Intercept $=7.224\;{cm}^{2}$ (95% CI 3.50–11.6; p<0.01 ), VAT slope =1.112 (95% CI 1.063–1.157; p<0.01 ), and Obesity $=7.583\;{cm}^{2}$ (95% CI 1.978–13.035; p=0.014 ). Neither sex nor age category nor any interaction term reached significance (all p>0.1 ). Model $R^{2}=0.978$ ; Bland–Altman LoA =−21.46 to $+21.51\;{cm}^{2}$ .

To adjust for the errors in automated SAT surface determination, we performed linear regression using the SAT surface calculated by the manual reference method as the dependent variable, while SAT surface calculated by the Otsu algorithm and age category (with childhood as reference) were selected as independent variables. The regression was statistically significant $\left(R^{2}=0.998;\;F=13902.99\;\left({\text{df}}_{\text{Regression}}=2;\;{\text{df}}_{\text{Residual}}=65\right);\;p<0.01\right)$ , and the fitted regression model was Equation 2.

$$\begin{array}{lll} {SAT}_{\text{manual}} & = & 0.67+1.037\times {SAT}_{\text{Otsu}}+3.337\times I_{\text{Adult}}, \end{array}$$ (2)

where all areas are in ${cm}^{2}$ and $I_{\text{Adult}}=1$ for adults and 0 for children. A more detailed overview of the regression model is provided in Supplementary Table S9. The model exhibited excellent correlation with the manually determined SAT surface $\left(R^{2}=0.998\right)$ , as displayed in Supplementary Figure S4. Bland–Altman analysis after applying Equation 2 to the Otsu results provided LoA ranging from −9.22 to $+9.31\;{cm}^{2}$ (Supplementary Figure S5). Refitting the WLS SAT model on the full cohort produced: Intercept $=0.67\;{cm}^{2}$ (95% CI −2.116 –3.179; p=0.622 ), SAT slope =1.037 (95% CI 1.020–1.056; p<0.01 ), and Age category $=3.337\;{cm}^{2}$ (95% CI 1.134–5.674; p<0.01 ). No other terms, including interactions, were significant (p>0.05) . $R^{2}=0.998$ ; Bland–Altman LoA =−9.22 to $+9.31\;{cm}^{2}$ .

Internal validation confirmed the stability of both correction models. In leave-one-out cross-validation, the VAT correction model achieved $R^{2}=0.976$ , RMSE $=11.45\;{cm}^{2}$ , MAE $=8.32\;{cm}^{2}$ , and mean bias $=0.15\;{cm}^{2}$ , whereas the SAT correction model achieved $R^{2}=0.997$ , RMSE $=5.03\;{cm}^{2}$ , MAE $=3.39\;{cm}^{2}$ , and mean bias $=0.21\;{cm}^{2}$ . Repeated 10-fold cross-validation (100 repeats) yielded nearly identical mean performance (VAT: $R^{2}=0.976$ , RMSE $=11.48\;{cm}^{2}$ , MAE $=8.35\;{cm}^{2}$ , mean bias $=0.15\;{cm}^{2}$ ; SAT: $R^{2}=0.997$ , RMSE $=5.07\;{cm}^{2}$ , MAE $=3.42\;{cm}^{2}$ , mean bias $=0.21\;{cm}^{2}$ ).

Pairwise correlations among sex, age category, and obesity status were modest (absolute r≤0.368 ). Variance inflation factors (VIFs) were low for the demographic predictors (1.12–1.26), the main-effects candidate correction models (VAT, 1.36–2.09; SAT, 1.16–2.12), and the final reduced correction models (VAT, 1.05 for both predictors; SAT, 1.09 for both predictors). In centered interaction-screening models, all VIFs remained below 2.5, indicating no evidence of problematic multicollinearity.

### 3.7 Clinical correlations

After applying the regression corrections to Otsu-derived VAT and SAT, per-patient areas $\left({cm}^{2}\right)$ were computed along with VAT/SAT ratios and VAT and SAT as percentages of TAT. We then compared these indices across sex, weight status, and diagnosis categories.

The VAT/TAT ratio was normally distributed in the overall cohort and within age groups, diagnosis types, sexes, and weight categories. In the full cohort, adults had significantly higher VAT/TAT percentages and VAT/SAT ratios than children. Overweight patients showed the highest VAT/TAT and VAT/SAT, followed by normal weight, with obese patients showing the lowest values (Supplementary Table S10).

When analyzing the group as a whole, adults had significantly higher VAT/TAT percentages and VAT/SAT ratios when compared to children. In addition, overweight patients had the highest VAT/TAT percentages and VAT/SAT ratios, followed by normal-weight patients, with obese patients showing the lowest values for these parameters. Further details concerning these statistically significant differences can be found in Supplementary Table S10.

When strictly considering the adult population of the study, VAT, SAT, and TAT surfaces showed significantly larger values with increasing weight categories. No correlation was found, however, between VAT/TAT percentage or VAT/SAT ratio and weight category, as presented Supplementary Tables S11, 12. Furthermore, in adult patients, there was a positive correlation between the absolute values of VAT, SAT, and TAT surfaces with BMI (as shown in Supplementary Table S13; Supplementary Figures S6-S8). In contrast, no significant correlation was found between BMI and adipose tissue proportion measured by VAT percentage from TAT and VAT/SAT ratio. Within the adult demographic, males demonstrated significantly larger VAT surfaces as well as greater VAT/TAT percentages and VAT/SAT ratios when compared to their female counterparts. With respect to the diagnosis type, adult patients exhibiting benign pathologies manifested significantly lower values for SAT and TAT surfaces.

## 4 Discussion

This research was based on MRI images acquired under standard clinical protocols from a diverse cohort spanning demographic, anthropometric, and clinical profiles. Our primary objective was to develop and validate a freely available, open-source algorithm for fully automated adipose tissue segmentation. While the present pipeline was initially designed for single-slice analysis at the L2 level for ease-of-use and simplicity, its modular structure allows straightforward extension to multi-slice or fully volumetric MRI data. In a multi-slice implementation, the same preprocessing, segmentation, and VAT/SAT delineation procedures can be applied independently to each axial image within a contiguous stack, after which volumetric measurements can be obtained by aggregating slice-wise areas using voxel spacing and slice thickness derived directly from DICOM metadata, similar to previously described algorithms (Shen et al., 2004b) (Equation 3):

$$V=\left(t+h\right)\overset{N}{\underset{i=1}{\sum }}A_{i},$$ (3)

where V is the calculated volume, $A_{i}$ is the cross-sectional area of each scan (i.e., VAT, SAT, or TAT as determined by the pipeline), h is the between-slice interval, t is the thickness of each slice, and N is the total number of slices.

Importantly, no structural modification of the segmentation algorithm is required, as the pipeline operates independently on each slice and can therefore be applied sequentially to volumetric datasets. Multi-slice and volumetric MRI approaches may improve robustness by reducing sensitivity to slice misplacement and more accurately capturing the heterogeneous cranio–caudal distribution of adipose tissue. However, these methods require precise handling of voxel geometry and a consistent definition of anatomical boundaries to ensure reliable volume estimation. In this context, automated vertebral recognition algorithms may be particularly useful, as they enable reproducible localization of anatomically standardized regions across subjects and imaging sessions, thereby improving consistency in volumetric adipose tissue quantification (Zhao et al., 2021). We compared the efficiency of Otsu thresholding, k-means, and fuzzy C-means for adipose–lean discrimination, using a validated manual segmentation reference for performance assessment (Maddalo et al., 2017). Across the full cohort, Otsu thresholding yielded the best overall agreement with manual reference, with mean Dice coefficients of 0.9647±0.0150 for SAT, 0.9152±0.0333 for TAT, and 0.7863±0.1070 for VAT. K-means was consistently second and fuzzy C-means third; although differences were statistically significant, they were numerically small. These trends persisted in subgroup analyses, with higher Dice values observed in adults and in males, and with performance improving with increasing BMI. The cluster analysis further supported this pattern, showing a monotonic rise in VAT Dice from cluster 1 (predominantly normal-weight female children) to cluster 5 (predominantly overweight/obese adult males). We further introduced a simple, accurate rule for demarcating visceral (VAT) from subcutaneous adipose tissue (SAT). Compared with existing approaches, both manual and automated, our method balances computational efficiency with implementation simplicity. Traditional manual segmentation remains a common reference for VAT/SAT delineation despite being time-consuming (Pescatori et al., 2019; Poonawa et al., 2013) and is still treated as a standard for benchmarking automated methods (Poonawa et al., 2013; Maddalo et al., 2017); its enduring value stems from its accuracy, even as automated approaches gain adoption.

Automated VAT/SAT separation spans several families: (1) *Region growing* (Thörmer et al., 2013), which offers flexibility but requires careful seed selection and threshold criteria and can be error-prone or time-intensive (Poonawa et al., 2013); (2) *Active contours* (Positano et al., 2004; Thörmer et al., 2013; Würslin et al., 2010), which iteratively refine curves to lock onto image features such as edges (Samara et al., 2012), including accelerated variants like extended snakes with balloon forces (Positano et al., 2004); and (3) *Morphological operators/shape models* for elliptical approximation of VAT/SAT boundaries (Maddalo et al., 2017). Specific implementations include elliptical fitting of the inner SCAT contour with morphological post-processing (Brown et al., 2015), gradient-magnitude maps with orthogonal convex-hull tools (Eloi et al., 2017), and adjustable contour constraints (Samara et al., 2012). While adaptable and sometimes high-performing, these methods can be complex and time-consuming compared with our streamlined approach designed to be robust and efficient.

Although the thresholding and clustering components used here are classical rather than state-of-the-art AI architectures, the contribution of the present work lies in integrating them into a fully automated, interpretable pipeline tailored to routine opposed-phase T1-weighted single-slice MRI, together with a simple centroid-based VAT/SAT separation rule and regression calibration of systematic bias. Recent deep-learning studies for abdominal adipose tissue segmentation on Dixon or water-fat MRI have generally reported strong internal segmentation metrics, with subcutaneous adipose tissue Dice values often around 0.90–0.99 and VAT Dice values around 0.83–0.98, frequently with excellent agreement for volume or area estimation on internal validation (Estrada et al., 2020; Kway et al., 2021; Bhanu et al., 2022; Ogunleye et al., 2023; Wu et al., 2024; Kafali et al., 2024). However, these models were typically developed on Dixon, water-fat, or other dedicated volumetric MRI acquisitions and relied on expert-annotated training data, ranging from smaller manually labeled datasets to substantially larger cohort-scale collections (Estrada et al., 2020; Küstner et al., 2020; Kway et al., 2021; Bhanu et al., 2022; Ogunleye et al., 2023; Wu et al., 2024; Kafali et al., 2024). Related work has also shown that fully convolutional networks can achieve operator-level performance for full abdominopelvic adipose quantification in patients with obesity (Schneider et al., 2023). In contrast, our training-free method achieved mean Dice coefficients of 0.9647 for SAT, 0.9152 for TAT, and 0.7863 for VAT, with corresponding Hausdorff distances of 9.20, 10.72, and 7.83, respectively. Weighted least-squares regression calibration further improved quantitative area estimation, with excellent full-cohort agreement against manual measurements (VAT: $R^{2}=0.978$ ; SAT: $R^{2}=0.998$ ), and this performance remained stable under internal validation by leave-one-out and repeated 10-fold cross-validation. We therefore view the present approach not as a replacement for state-of-the-art deep learning on harmonized Dixon datasets, but as a pragmatic, transparent, and open-source alternative for settings in which large annotated datasets, standardized water-fat imaging, or dedicated AI infrastructure are not available. A direct head-to-head benchmark on matched data would nevertheless be an important next step. We also examined how patient-level factors influence accuracy for the best-performing method. Results align with prior literature on adipose distribution across sex and age (Shen et al., 2009). Our models tended to perform better with increasing BMI and showed superior outcomes in adults and men. This is clinically relevant for cardiovascular risk profiling, as age and male sex are traditionally associated with elevated risk (Kuk et al., 2005; Han et al., 2023), an association compounded by obesity—particularly when perivisceral adiposity is prominent (Lemieux and Després, 2020). Midlife obesity in men has also been linked to later-life quality-of-life decline (Anderson et al., 2022). These observations suggest overweight adult males may be prime candidates for risk stratification using VAT quantification.

At the same time, women are frequently underdiagnosed and undertreated for cardiovascular disease, partly due to assumptions about premenopausal cardioprotection; nevertheless, they remain vulnerable to higher mortality and poorer outcomes after cardiovascular events (Kuk et al., 2005). These disparities underscore the need for targeted diagnostic and preventive strategies for women. Additionally, extending adipose tissue quantification to children may inform early preventive interventions aimed at reducing adult cardiovascular burden associated with childhood risk factors (Müller et al., 2016).

In our data, Otsu thresholding yielded the most accurate overall results, and we used this method to quantify abdominal adipose tissue disposition. The cohort spanned a wide age range, similar to earlier work (Shen et al., 2009; Kuk et al., 2005), and included heterogeneous pathologies, including malignancies, where adipose quantification has received focused attention (Han et al., 2023; Anderson et al., 2022). Consistent with prior reports, patients with cancer and cachexia or substantial weight loss exhibited lower VAT and SAT areas than healthy controls or non-cachectic cancer patients. Han et al. (Han et al., 2023) also reported increased VAT in benign colorectal disease versus healthy participants or colorectal cancer with/without cachexia (Han et al., 2023). In our study, pathology-wise comparisons showed decreased TAT and SAT in benign conditions, with no significant change in VAT. Our aim was to deliver segmentation that remains reliable across varied clinical contexts. Because Otsu segmentation systematically underestimated areas, we derived bias-correction models with excellent fit to manual measurements. VAT was, on average, underestimated by $20.03\;{cm}^{2}$ (SD $14.06\;{cm}^{2}$ ); SAT by $9.22\;{cm}^{2}$ (SD $5.81\;{cm}^{2}$ ). The weighted least-squares VAT model in Equation 1 and the SAT model in Equation 2 corrected these biases, achieving $R^{2}=0.978$ and $R^{2}=0.998$ , respectively, and Bland–Altman limits of agreement of −21.46 to $+21.51\;{cm}^{2}$ for VAT and −9.22 to $+9.31\;{cm}^{2}$ for SAT. From a clinical perspective, the observed systematic underestimation represents a consistent measurement offset rather than random error. Because this bias was predictable and could be effectively corrected using regression-based adjustment, it did not compromise the ability of the automated method to distinguish differences between groups, assess adipose tissue distribution, or support comparative and longitudinal analyses. After correction, adults exhibited higher VAT/TAT% and VAT/SAT than children; overweight participants showed the highest VAT/TAT% and VAT/SAT, followed by normal weight, and then obesity. In adults, absolute VAT, SAT, and TAT areas increased with BMI, whereas VAT/TAT% and VAT/SAT did not; adult males had larger VAT areas and higher VAT/TAT% and VAT/SAT than females.

As expected, VAT, SAT, and TAT areas increased by BMI category, and in adults each correlated positively with BMI. However, adipose disposition indices (VAT/TAT% and VAT/SAT) did not mirror these correlations in adults. When analyzing the full cohort, overweight participants exhibited the highest VAT/TAT% and VAT/SAT. This heterogeneity likely reflects a known limitation of BMI as a sole obesity metric—it does not capture body-composition differences (Müller et al., 2016). Men in our sample showed a higher propensity to obesity and, among adults, a stronger tendency toward visceral predominance; this pattern agrees with established sex dimorphism in adiposity (Palmer and Clegg, 2015). Adults overall were more prone to increased visceral deposition, even though obesity was more prevalent in children within our dataset. This may help pinpoint when obesity transitions toward its more deleterious visceral phenotype and motivates further study. Given that VAT and SAT surface areas in abdominal MRI span a wide range depending on patient age, sex, and weight status, the limits of agreement identified in the Bland–Altman analysis suggest that the automated method provides measurements with a level of accuracy suitable for adipose tissue quantification in both research and clinical contexts. In particular, this method is well adapted for clinical applications such as risk stratification, population-level analysis, and longitudinal monitoring, which focus on broader trends and relative differences rather than small absolute variations. In this context, the observed level of agreement, together with the strong correlation between automated and manual measurements, supports the use of the automated method as a reliable tool for adipose tissue assessment.

This study has limitations, notably a modest sample size and cohort heterogeneity inherent to retrospective single-center data. Even so, the algorithm produced results consistent with known relationships and provides a practical framework for evaluating classical adipose quantification methods. We used bivariate analyses, clustering, and linear regression to identify patient-trait patterns associated with varying algorithm accuracy and leveraged these insights to correct Otsu-based VAT and SAT surfaces via linear models (see Equation 1; Equation 2). These calibration equations were derived from a single-center cohort using a specific opposed-phase T1-weighted MRI protocol and a single L2 slice, and should therefore be interpreted as cohort- and protocol-specific rather than universally transferable. Although internal cross-validation showed stable performance of the correction models, this does not substitute for true external validation. Accordingly, the VAT and SAT correction equations should be independently validated in future studies and, if necessary, recalibrated before use in datasets acquired with different scanners, sequence parameters, slice selection strategies, or patient populations.

## 5 Conclusion

Our streamlined classical image-processing pipeline, enhanced by a simple VAT/SAT delineation rule and regression-based error correction, enables accurate abdominal fat quantification from a single opposed-phase MRI slice using standard clinical data. This method is advantageous for large-scale studies and for assessing incidental cardiovascular and metabolic risks in both adult and pediatric populations, all without the need for extra scan time or specialized acquisition protocols. The publicly available code and models serve as a solid foundation for extending our approach and facilitating broader adoption in research and, ultimately, clinical practices.

## Data Availability

The datasets presented in this study can be found in online repositories. The names of the repository/repositories and accession number(s) can be found below: https://github.com/bogdanneamtu76/Adipose_Tissue_Segmentation.
